# Two dramatically different clinical scenarios of neonatal Echovirus-11 infection in late preterm male twins: a case report and review of the literature

**DOI:** 10.1186/s13052-025-01880-5

**Published:** 2025-02-20

**Authors:** Simona Perniciaro, Caterina Proietti, Angela Bossi, Roberta Maragliano, Carla Facco, Federica Novazzi, Nicasio Mancini, Massimo Agostino Agosti

**Affiliations:** 1 Neonatal Intensive Care Unit, “Filippo del Ponte” Hospital, ASST Settelaghi, Varese, 21100 Italy; 2 Department of Medicine and Surgery, Unit of Pathology, University of Insubria, Varese, Italy; 3 Faculty of Medicine and Surgery, Department of Medicine, University of Insubria, Technological Innovation Varese, Varese, IT Italy; 4 ASST Sette Laghi, Laboratory of Medical Microbiology and Virology, Varese, IT Italy; 5 Department of Medicine and Surgery, University of Insubria, Varese, IT Italy

## Abstract

**Background:**

Enterovirus is a well-known cause of infection in all age groups, with particular importance for neonates with both vertically and horizontally transmission. Neonatal clinical manifestations are highly variable and mostly is asymptomatic, but severe infections are described such as myocarditis, meningitis, encephalitis, hepatitis, coagulopathy, pneumonia and viral sepsis. Since 2022, The World Health Organization has recently reported an increasing number of severe neonatal infection associated with a new variant of Echovirus-11 (E-11). Many of the infants described with E-11 infections in a case reports series were preterm, male and twins. Despite the criticality of neonatal disease, the clinical management remains primarily supportive and therapeutic options are unfortunately few.

**Case presentation:**

We present the case of male dichorionic diamniotic late-preterm twins, born in December 2023 at Filippo del Ponte Hospital in Varese (Northen Italy) with early Echovirus-11 infection. They had two dramatically different clinical scenarios and one of them developed a severe and fatal hemorrhage-hepatitis syndrome.

**Conclusions:**

Our experience has the purpose to emphasize public health attention to Echovirus-11 neonatal infections and their rare dramatically neonatal clinical presentations. Prematurity, host genetic predisposition and vertical transmission seem to be strong risk factors for severe infections, but it still need to be elucidated. According to previous Italian report, a surveillance protocols in all cases with unexpected clinical presentations and sequencing complete genome in order to better understand typing and molecular characterization of emerging and re-emerging pathogenic variants and new strains are strongly recommend in Italy.

## Background

Enterovirus is a well-known cause of infection in all age groups, with particular importance for neonates [1]. Its transmission to the newborn may occur both vertically and horizontally, including occasional nosocomial transmission. Clinical manifestations are highly variable and, in about 50% of cases, the infection is asymptomatic. Myocarditis the manifestation with the highest lethality rate and is often associated to coxsackievirus B infection [2]. Severe neonatal infections are also characterized by hepatitis and coagulopathy, recently associated to a new variant of Echovirus-11 (E-11) [2, 3].

The early vertically-acquired neonatal infection usually manifests itself within the first seven days of life and is sometimes associated with antenatal fever or mild moderate gastrointestinal symptoms in the mothers [2].The World Health Organization (WHO) publicly launched an alarm on the worrying situation of the rising number of severe neonatal infections related to E-11 [4]. Despite the low public health risk for the general population, the WHO is concerned about the increase in reported cases. This issue is also well-known in Europe and The European Centre for Disease Prevention and Control (ECDC) has recently included this virus in the ECDC Communicable Disease Threat Report [5].

We present the case of late preterm male twins with E-11 infection who had two opposite outcomes.

Clinical data was obtained from medical and pathological record and genotyping of virus has been requested from our Virology Department and was performed by whole genome sequencing (WGS).

PubMed and Web of Science databases were searched for reviews on neonatal Echovirus-11 infection (e.g. Echovirus-11, Enterovirus, neonates, vertical infection, hemorrhage-hepatitis syndrome, twins, male sex, host genetic predisposition).

Informed consent has been requested from the parents for the publication of clinical data

## Case presentation

We describe the case of male dichorionic diamniotic (DCDA) twins, born in December 2023 at Filippo del Ponte Hospital in Varese (Northen Italy). The infants were born at 35 + 5 weeks of gestation, delivered by emergency cesarean section because unstoppable labor in preterm rupture of membranes. First twin (CASE B) had a birth weight of 2000 g (with a growth percentile of 10°−25°) and second twin (CASE A) weighed 2710 g (with a growth percentile of 64°) at birth. Both had Apgar scores of 9 and 10 at 1 and 5 min, respectively.

In the last days before giving birth, the mother later reported she had suffered a mild rhinitis rapidly resolved. Because both neonates did not have signs of infection, the initial practice of watchful waiting was adopted.

### Case A

After an initial good adaptation, the infant developed a mild respiratory distress and he was transferred to Neonatal intensive care unit (NICU) at 9th hour of life. Respiratory support with High Flow Nasal Cannula (HFNC) was started (maximum flow 7 l/min, FiO2 maximum 0,30) and blood investigation was performed with no sign of laboratory alteration, except for asymptomatic hypoglycemia. X-ray done on day 1 showed bi-basal accentuation of lung interstitium compatible with transient tachypnea of the newborn. On day 2 for the persistent mild respiratory distress, blood culture was performed first and then antibiotic therapy with ampicillin and amikacin was started in the suspicion of neonatal sepsis. After 48 h from the beginning of the therapy, the infant appeared to be slightly improved and antibiotics were stopped with negative blood culture and infection bio-markers.

On the 6th day of life, the infant was still supported with HFNC and episodes of apneas was noted, infection biomarkers were repeated and still unremarkable.

On day 7, a few hours later the infant became lethargic with increased oxygen requirement, arterial blood results showed metabolic acidosis (BE-12,4, Lac 4 mmol/l), elevated white blood cell (WBC 32.94 10^9/l), severe thrombocytopenia (17 × 10^9/l) and coagulopathy (PT ratio 2,37, aPTT ratio: 4,67, fibrinogen 62 mg/dl). Antibiotic treatment with ceftazidime and vancomycin plus antiviral acyclovir were started, infusion of platelets and fresh frozen plasma (FFP) have been done. He was rapidly intubated and mechanically ventilated.

Laboratory testing was consistent with acute hepatitis with elevated liver enzymes and mild renal dysfunction (creatinine 1.39 mg/dl, urea 53 mg/dl), severe coagulation disorders and anemia. The significant laboratory values are reported in Table 1.

**Table 1:** Significant blood investigations from Day 7th to Day 9th

Blood investigations	Day 7th	Day 8th	Day 9th
***WBC 10^*** ^***9***^ ***/l***	32.94	32.22	37.12
***Hemoglobin g/dl***	10.8		10
***Platelets 10^*** ^***9***^ ***/l***	17	161	75
***PT-INR***	2,37	5.25	5.19
***aPTT***	4,67	2.37	1.67
***Fibrinogen mg/dl***	62	91	90
***Albumin g/dl***	2.3		2.8
***AST U/L***	--	> 7000	--
***ALT U/L***	--	729	523
***Troponin ng/L***	363	279	--
***Ammonia µg/dl***	--	346	--

Lumbar puncture was performed, cerebrospinal fluid (CSF) WBC count was 20 cells/µL, protein concentration was 61 mg/dl and CSF glucose was 65 mg/dl. The FILMARRAY™ Meningitis/Encephalitis test (bioMerieux, Marcy l’Ètoile, France), a multiplex real-time PCR assay for the detection of several pathogens in CSF, showed positivity for Enterovirus. Further virological testing confirmed the presence of enteroviral RNA by reverse transcriptase-polymerase chain reaction (RT-PCR) also in plasma (676.241 RNA copies/ml) and on rectal sample (83808 RNA copies/ml).

The infant’s clinical condition dramatically deteriorated, he underwent intensive daily transfusion treatment including 8 times FFP and 5 times platelet units, red blood cells (on days 7), intravenous immunoglobulins (IGIV) on day 10 and 2 times albumin transfusion. Inotropic support (dopamine◊noradrenalin) and hydrocortisone were mandatory due to viral septic shock with severe hypotension, maximum troponin value was 363 ng/L. Serial echocardiography showed cardiac hyperkinesis without myocardial dysfunction or pericarditis.

The clinical presentation was mainly characterized by fulminant hepatitis and severe coagulopathy, on day 9 alanine transaminase (ALT) decreasing to 523 U/L with no sign of normalization of coagulation despite multiple transfusion (PT ratio 4,83; aPTT 1,67, fibrinogen 90 mg/dl).

On day 10 a severe pulmonary hemorrhage has occurred and this quickly resulted in serious respiratory failure that led to patient’s death.

**Fig. 1:**
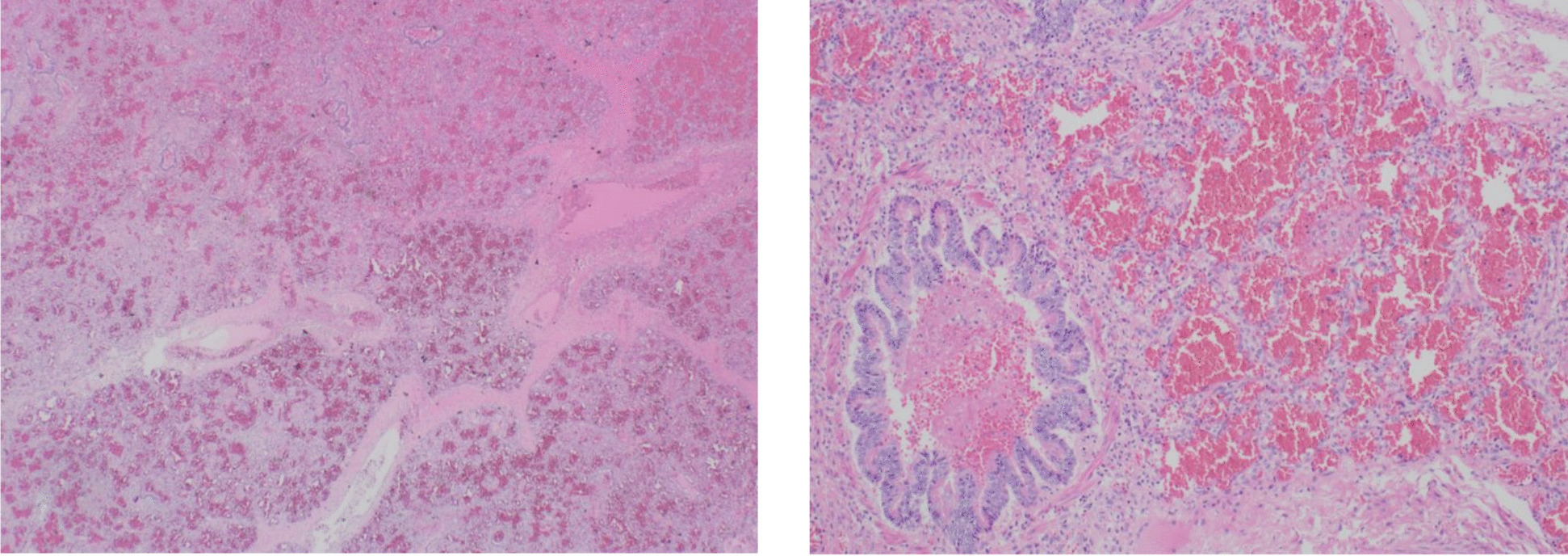
Pulmonary hemorrhage ​with diffuse alveolar hemorrhage. The alveoli are filled with extravasated erythrocytes (hematoxylin and eosin stain, 10x magnification)​

**Fig. 2:**
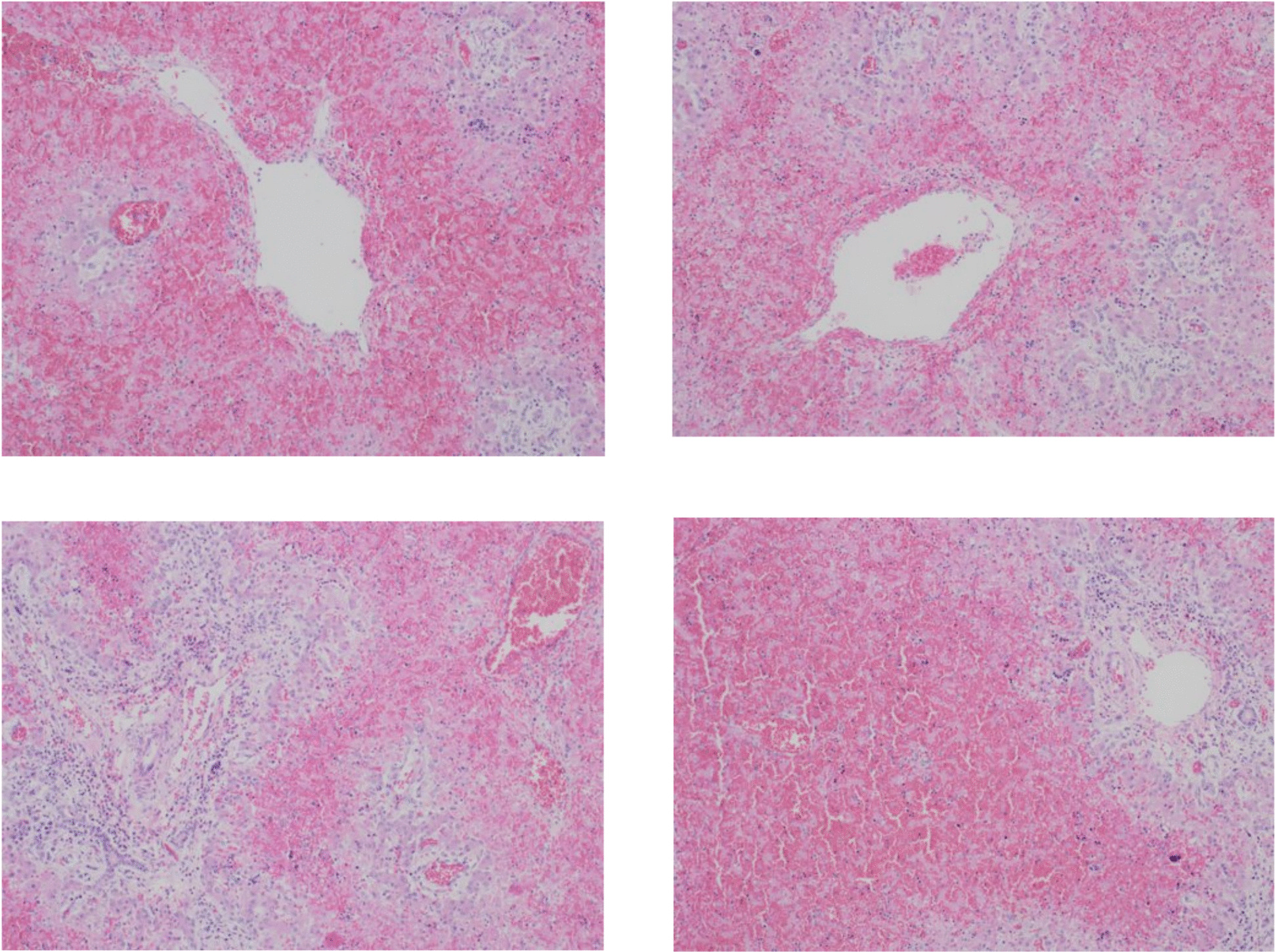
Massive hepatic necrosis with remaining periportal hepatocytes. Foci of confluent necrosis across multiple lobules are present. (hematoxylin and eosin stain, 10x magnification)

**Fig. 3:**
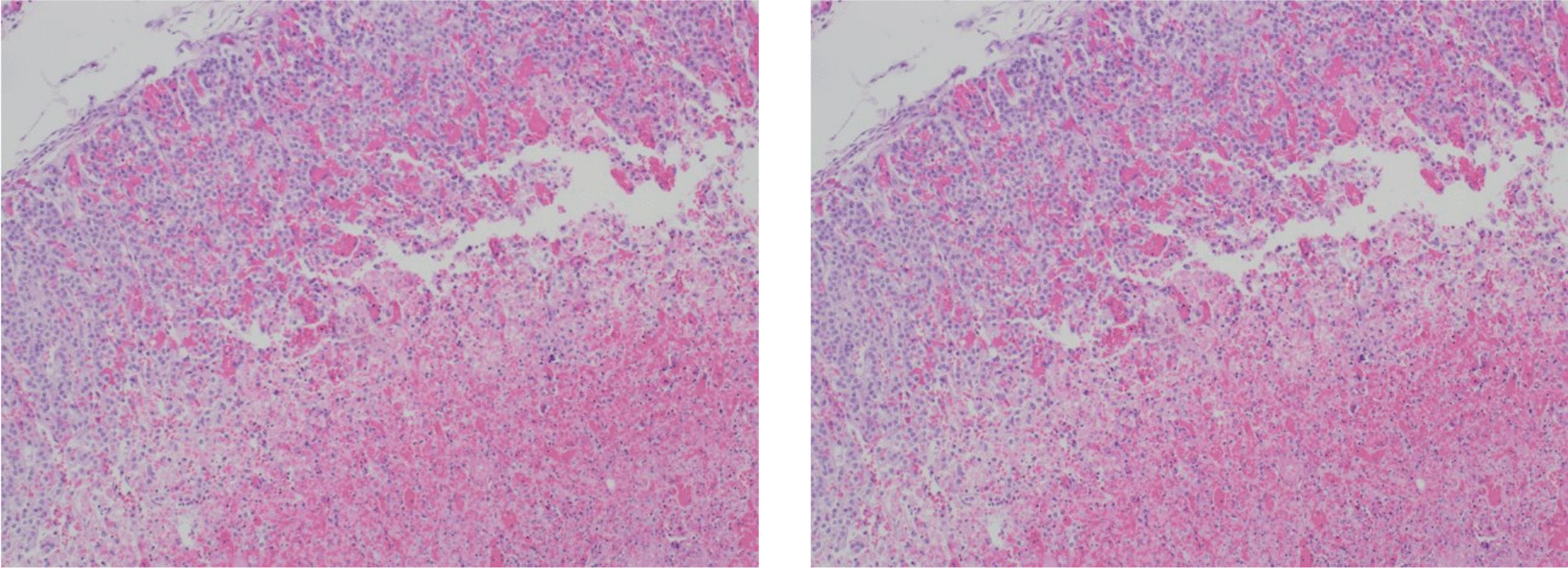
Adrenal hemorrhage (hematoxylin and eosin, 10x magnification)

**Fig. 4:**
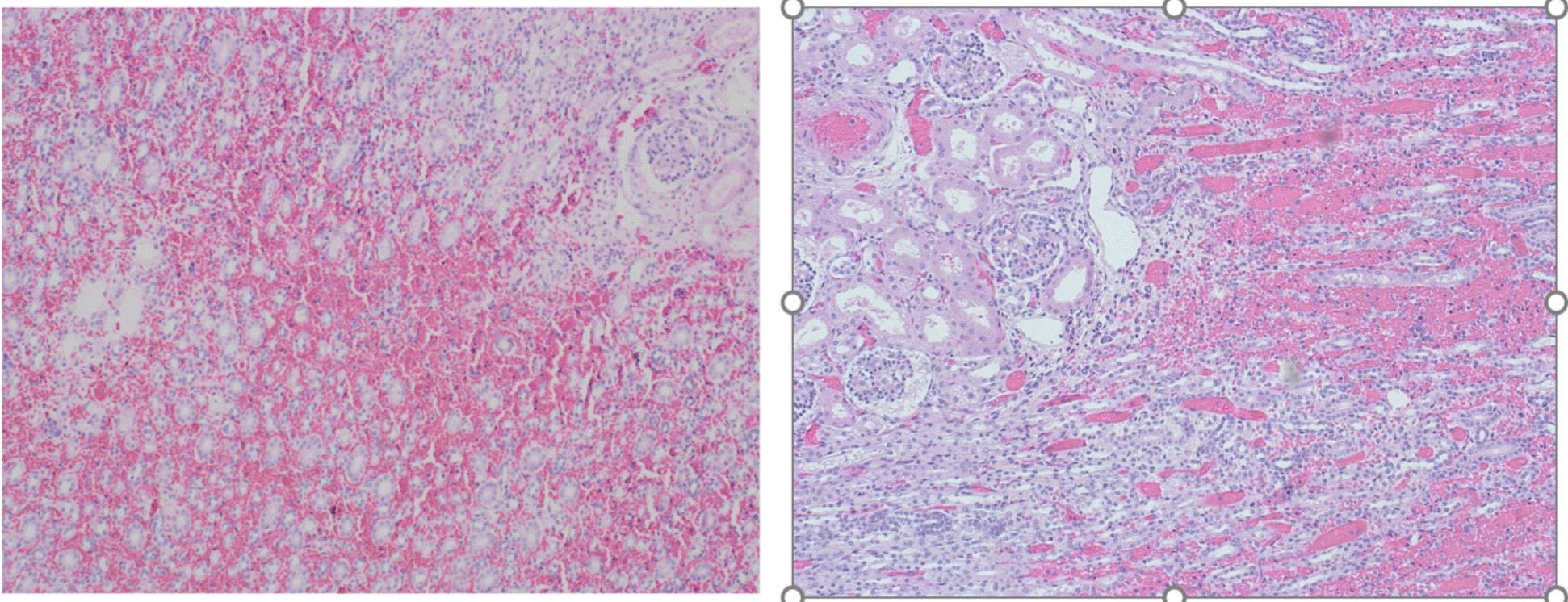
Renal medullary hemorrhage (hematoxylin and eosin, 10x magnification)

Postmortem examination showed centrilobular massive hepatic necrosis and bilateral pulmonary, renal and adrenal glands hemorrhage with pericardial, pleural and peritoneal effusion (Figs. 1, 2, 3 and 4).

Genotyping of Enterovirus has been requested from our Virology Department and was performed in plasma and CSF by WGS and showed the presence of Echovirus-11 strains.

### Case B

On day 7 of life, the second twin was still asymptomatic.

However, because of the diagnosis of severe neonatal viral sepsis like syndrome with fulminant hepatitis of his brother, the infant received a special serial clinical observation but rooming-in was still promoted. RT-PCR of rectal and pharyngeal swabs was performed and Enterovirus were detected with high viral load. (5577 RNA copies/ml and 1.708.215 RNA copies/ml respectively). Because of persistent well appearing, limited blood investigation was done (blood count, renal and liver function) and returned normal. Echo cerebrum did not show any altered signs. After 11 days of observation the infant has been discharged in good general health conditions. He has always maintained a clinical well-being and regular growth confirmed in regular follow-up visits. Rectal and pharyngeal swabs returned negative at 13 day of life.

Enterovirus typing was performed in rectal and pharyngeal swabs by WGS and showed the same presence of E-11 strains.

After the newborn’s positive tests, the mother performed a negative nasal swab test, while the vaginal swab test was positive for Echovirus-11 by WGS.

This confirms the probable vertical transmission from the mother to newborns.

## Discussion and conclusions

### Epidemiology

Enteroviruses belong to the genus *Enterovirus*, family Picornaviridae and are some of the most common viral pathogens in young childrens [6]. According to the International Committee on Taxonomy of Viruses classification, human enteroviruses can be classified into 12 species and include different serotypes: EV-A, EV-B, EV-C, EV-D, EV-E, EV-F, EV-G, EV-H EV-I, EV-L, EV-J, EV-K and EV-L [7].

Enteroviruses circulate worldwide, with some key regional differences between species [8]. For example, the prevalence of EV-C is highest in Africa, and EV-A particularly high in Asia [8]. Enterovirus infections have a significant impact globally and suggest that there is a wide variety of enterovirus types [8].

According to the data from Centers for Disease Control and Prevention in America, non-polio enteroviruses cause 10–15 million infections and tens of thousands of hospitalizations in the United States each year [9]. Infections is typically seasonal, and the peak of disease occur in the summer and early fall in regions with a temperate climate around the globe [10].

### Clinical presentation

Enteroviruses are mainly transmitted by fecal–oral and respiratory routes and result in a wide range of clinical outcomes, from asymptomatic infection that account for approximately 50% of the cases to symptomatic infections ranging from nonspecific febrile illnesses to life-threatening diseases such as myocarditis, meningitis, encephalitis, hepatitis, coagulopathy, pneumonia and viral sepsis. The main manifestations include hand-foot-mouth disease, acute hemorrhagic conjunctivitis and herpangina, as is well known [11]. The greatest morbidity are in children younger than 5 years and infants are the most at risk of severe infections [12, 13].

In neonates Enterovirus may be acquired vertically before, during, or after delivery (exposure to maternal blood, secretions, and/or stool) and horizontally from family members [14]. Nosocomial transmission in nurseries or in NICU are rare but have been described [15].

Occasionally enteroviruses may cause large-scale outbreaks or continuous epidemics in NICU. For example, in April 2019, a potential nosocomial infection was reported from a neonatal department in Guangdong (China). The infection cluster was associated with five deaths, which tested positive for Echovirus-11 by Multiplex real-time RT-PCR [16].

Transmission by human milk during breastfeeding has been described as well [17].

In neonates, Enteroviruses can seriously affect the cardiovascular and the nervous system, resulting in myocarditis, meningoencephalitis and other severe complications. Because of the functionally different immune system, newborns are at high risk for the development of serious clinical manifestations of infectious disease [18].

Only 21% of infected newborns have symptoms and just a minor subset develops severe illness. The lack of systematic diagnosis makes it difficult to estimate the real incidence and morbidity of human enteroviruses infection in this age group and they are sometimes confused with bacterial sepsis [19].

Zhang et al. conducted a systematic review on severe enterovirus infections in 237 neonates. Most cases (70.5%) exhibited symptoms within the first 7 days of life, 6.8% reported siblings or friends with symptoms and 29.5% were associated with maternal disease before delivery suggesting the lack of specific transplacental neutralizing antibodies against the infecting serotype in newborns. Sex and mode of birth were not significant [2].

Enterovirus was identified mostly from the rectal or stool samples (118, 49.8%), followed by respiratory samples (101, 42.6%). Additionally, most of infections were caused by coxsackievirus B (82.7%), followed by echovirus (16,7%).The clinical manifestations included temperature abnormalities (53.6%), rash (37.1%), poor feeding (24.5%), respiratory symptoms (19.0%), lethargy (16.9%), jaundice (16.0%), circulatory failure or shock (15.6%), arrhythmias (12.2%), thrombocytopenia (11.8%), poor perfusion (9.7%), irritability (7.2%), hypotonia (3.8%), and diarrhea (3.8%) [2].In almost 50% of reported cases complication was hepatitis or coagulopathy and frequently was associated with myocarditis, hypertrophic cardiomyopathy, intracranial hemorrhage, encephalitis, disseminated intravascular coagulation, renal injury and pneumonitis. In neonates with hepatitis mortality rate was high (26,6%) and cases of survivors that required liver transplantation are described [2].

Myocarditis is the second major complication with severe outcomes and highest lethality rate of 38.6% and 40,7% of heart sequelae in the survivors [2].

Other rare complications include meningoencephalitis, hemophagocytic lymphohistiocytosis (HLH), pulmonary hemorrhage, persistent pulmonary hypertension, bone marrow failure and congenital skin lesions [2].

Specifically concerning Echovirus-11, an investigation of enterovirus circulation in European countries between 2015 and 2017 highlighted the wide circulation of non-polio enteroviruses in Europe and emphasized E-11 among the five most notified from children aged under three months [20]. In neonates, E-11 has been found to be more frequently associated with infection and death outcomes than other enteroviruses in the same population [20].

The WHO has recently reported an increasing number of severe neonatal infection associated with Echovirus-11 [4].

Since 2022, and as of 17 July 2023, 19 neonates with severe E-11 infection have been reported in the European countries, by France, Croatia, Sweden, Spain, and Italy, and nine of these neonates have died [21]. The United Kingdom reported two cases of E-11 infection in one pair of twins in March 2023. Both cases presented with several clinical conditions, including hepatitis and multiorgan failure, with rapid deterioration from the fourth day of birth to their death on the tenth day [21].

Between July 2022 and April 2023, nine cases of severe neonatal infection with a liver failure and neurological or myocardial involvement were reported in France. Seven of these children died. All were associated with a new variant of E-11. All cases were male: four pairs of premature twins and a full-term singleton. All presented clinical signs at three to six days old and maternal clinical symptoms, such as fever and gastrointestinal signs, were reported in four of five mothers during the three days before or on delivery [22].

Another report that is really similar to our, it has been reported recently in June 2023 by another Italian group; two cases of fulminant hepatitis linked with E-11 infection in late pre-term twin brothers. The mother presented with a single episode of fever at 35 weeks and two days of gestational age. The neonates have been transferred to a NICU and one of them has developed fulminant hepatitis and has been evaluated for liver transplantation. The phylogenetic and molecular analysis concluded that the Italian E-11 strains clustered with French strains collected in 2023 [23].

Again, public health authorities in Spain have reported two cases of severe E-11 infection in a couple of twins born in January 2023. Both cases were admitted to the NICU after birth with one recorded death and a diagnosis of severe enterovirus infection with probable vertical transmission, while the second case was discharged from the hospital without sequelae [24].

Further cases of E-11 infection have been reported in 2022 and 2023 in neonates, infants and older children, without full information of the clinical manifestations or outcomes. However, Austria, Belgium, Denmark, the Netherlands, Norway, and Portugal have not observed an increase of E-11 infections associated with severe neonatal cases [21].

Between August 1st 2021 and June 30th 2023, an epidemiological study of E-11 incidence in Italy showed a rise from the end of May 2023, which culminated at the end of June, which coincided with an increase in Enteroviruses-positive hospital cases. The E-11 identified belonged to the D5 genogroup and the majority (83%) were closely associated with the novel E-11 variant, first identified in severe neonatal infections in France since 2022. E-11 was identified sporadically in community cases until February 2022, when it was also found in hospitalized cases with a range of clinical manifestations. All E-11 cases were children, of these cases, 60% were neonates, and 71% had severe clinical manifestations [25].

The ECDC has recently included this virus in the ECDC Communicable Disease Threat Report and in order to better characterize the risk for neonates, ECDC asks Member States to provide information, through the online portal EpiPulse (Item ID 2023-EIP-00026) [21].

The risk of outbreak onset is well known, in 2018 an echovirus 11 outbreak in the neonatal intensive care units in a tertiary hospital in northern Taiwan has been described and authors have investigated the infection control efforts. The outbreak of Echovirus-11 in the NICU involved 10 neonates and was successfully controlled by the implementation of isolation, rapid surveillance and reinforced infection control measures [26].

Many of the infants described in a cases reports were preterm, evidence of a rise of Echovirus hemorrhage-hepatitis syndrome in neonates could be related with prematurity, low birth weight, premature rupture of fetal membrane, total parenteral nutrition (PN) (OR, 28.7; 95% CI, 2.8–295.1) and partial PN (OR, 12.9; 95% CI, 2.2–77.5) prior to the onset of disease [27].

Early onset of thrombocytopenia and decreased hemoglobin could be helpful in early identification of neonates with severe liver involvement. The low level of IFN-α and elevated expression of IP-10 may promote the progression of hemorrhage-hepatitis syndrome [27].

Prematurity, maternal history of illness, earlier age of onset ( ≦ 7 days), higher WBC (WBC ≧ 15,000/mm^3^) and lower hemoglobin ( ≦ 10.7 g/dL). are significant factors associated with hepatic necrosis with coagulopathy (HCN). Higher total bilirubin and concurrent myocarditis were most significantly associated with fatality from HNC that was up to 24% [28].

In another case series including 67 severe cases of neonatal enteroviral infection, the peak serum aspartate aminotransferase (AST) level was well correlated with the clinical outcome and patients with a serum AST level > 1000 IU/L, particularly > 2000 IU/L, had a significantly higher case-fatality rate [29].

Interesting evidence about prematurity and neonatal acute liver failure due to enteroviruses has been reported by Bambin Gesù Hospital in Rome, in fact in their long experience during 14 years data collection they reported 10 cases which were all preterm from 32 to 36 wks of gestations [30]. The reason why it is moderate or late prematurity that is a risk factor rather than severe prematurity is unclear.

Many of the reported cases were twins and high prevalence of boys in severe neonatal enterovirus infection might speak for a predisposition associated with X-chromosome [22].

### Diagnosis and therapy

The importance of early diagnosis is underlined by the work of K. Storm and colleagues that they have described a case report of female DCDA premature twins born at 35 weeks of gestational age with severe neonatal enterovirus infection with cardiac involvement, which proved fatal in one of the twins. The prompt identification in the other twin and early and aggressive supportive treatment with extracorporeal membrane oxygenation (ECMO) contributed to a positive outcome, emphasizing the importance of early recognition in averting adverse consequences [31].

Diagnosis of enterovirus infection is best achieved by viral RNA RT-PCR amplification on serum, pharyngeal secretions, stools, cerebrospinal fluid, urine or other materials and tissues. Abundant copies are present in the acute phase and viral shedding from the respiratory tract and feces continues for more than five weeks [19, 32].

Despite the criticality of neonatal disease, the clinical management remains primarily supportive and therapeutic options are unfortunately few.

Large doses of IGIV have been used in affected neonates showing favorable outcome and some authors suggests early administration within 3 days from illness onset [29, 33].

Pleconaril, an oral antiviral agent active on picornaviruses, has shown some potential. Pleconaril is a capsid inhibitor, which prevents the virus from attaching to cellular receptors and therefore prevents uncoating and subsequent release of viral RNA into the host cell. In a randomized trial of 61 neonates with suspected enterovirus disease who were assigned to seven days of oral pleconaril or placebo, there was a trend toward more rapid viral clearance (median 4 vs. 7 days) and lower overall mortality among pleconaril-treated infants (23 versus 44% with placebo). Thus, shorter times to culture and PCR negativity and greater survival among pleconaril recipients could support potential efficacy and warrant further evaluation. However, pleconaril is not currently available for systemic administration and larger trials are need in order to establish safety profile, proper regimens and clinical efficacy in reducing mortality and morbidity [34–36].

Another anti-viral drug called Pocapavir, is a potent, selective anti-enteroviral agent only available as an emergency investigational drug. The drug is a capsid inhibitor, administered orally and is highly protein-bound and excreted exclusively in feces. Pocapavir was used and successfully treated several severe cases of neonatal enteroviral infection, even with myocarditis [37–39].

It should be noted that both antiviral drugs are currently not available in Italy. Since neonates with severe enteroviral myocarditis may rapidly progress to cardiovascular collapse, which may be refractory to conventional medical treatment, ECMO may have a role in these patients [40].

Continuous veno-venous hemodiafiltration (CVVHDF) could be an extraordinary supportive treatment [41], especially in case of acute kidney injury, hyperammonemia and cardiopulmonary failure. In this study among children with severe hand, foot and mouth disease, CVVHDF group had significantly lower levels of these inflammatory factors, reduce heart rate and venous blood lactic acid and improve heart function than the conventional treatment group (*P* < 0.01)[42].

Immunomodulatory drugs (Etoposide, Cyclosporine A) and corticosteroid may be used when HLH is diagnosed [43]. Neonatal HLH related to infections account for 26% of cases, but enterovirus-Associated HLH remains extremely rare [44].

In a recent report about Enterovirus-Associated HLH the authors reported the hypothesis of addition of Anakinra to IVIG and Corticosteroids [45].

Anakinra, an interleukin-1-receptor antagonist, already used to treat pediatric and adult macrophage activation syndrome proving to be a successful treatment [46, 47].

Early detection and promptly supportive treatment seem to be the key to increased neonatal survival, although individual differences determine different outcomes.

## Conclusions

Our experience in accordance with data of recent E-11 cases reported has the purpose of underlining public health attention to non-polio enteroviruses and their rare and sometimes dramatically neonatal clinical presentations. The risk factors for a severe neonatal course are interesting topics of study and the underlying causative mechanisms still need to be elucidated. Prematurity, a host genetic predisposition in male and twin categories and probably the vertical transmission seems to be strong risk factors. The reported cases as well as our experience underline the enormous variability of outcomes in infected infants and host genetic factors affecting immunity might also influence the clinical severity [48].

This report highlights the need to test neonates for Enterovirus infection in a case of suspected viral sepsis or unclear neonatal infectious disease. According to previous Italian report, a surveillance protocols in all cases with unexpected clinical presentations and sequencing complete genome in order to better understand typing and molecular characterization of emerging and re-emerging pathogenic variants and new strains are strongly recommend in Italy [23].

## Data Availability

Available upon request to the corresponding author.

## Abbreviations

E-11: echovirus-11
WHO: World Health Organization
ECDC: European centre for disease prevention and control
DCDA: dichorionic diamniotic
WGS: Whole genome sequencing
HFNC: High flow nasal cannula
FFP: Fresh frozen plasma
ALT: Alanine aminotransferase
AST: Aspartate aminotransferase
NICU: Neonatal intensive care unit
PN: Parenteral nutrition
HCN: Hepatic necrosis with coagulopathy
ECMO: Extracorporeal membrane oxygenation
IGVI: Intravenous immunoglobulin
CVVHDF: Continuous veno-venous hemodiafiltration
HLH: Hemophagocytic lymphohistiocytosis
